# Causal Association Between Major Depressive Disorder and Cortical Structure: A Bidirectional Mendelian Randomization Study and Mediation Analysis

**DOI:** 10.1111/cns.70319

**Published:** 2025-03-09

**Authors:** Hui Zheng, Yong‐Jiang Fang, Xiao‐Ying Wang, Si‐Jia Feng, Tai‐Chun Tang, Min Chen

**Affiliations:** ^1^ The Acupuncture and Tuina School Chengdu University of Traditional Chinese Medicine Chengdu City China; ^2^ Department of Acupuncture Kunming Municipal Hospital of Traditional Chinese Medicine Kunming City China; ^3^ Department of Colorectal Diseases Hospital of Chengdu University of Traditional Chinese Medicine Chengdu China

**Keywords:** cortical structure, C‐reactive protein, inflammatory cytokines, major depressive disorder, Mendelian randomization

## Abstract

**Background:**

Previous observational studies have reported a possible association between major depressive disorder (MDD) and abnormal cortical structure. However, it is unclear whether MDD causes reductions in global cortical thickness (CT) and global area (SA). Objective: We aimed to test the bidirectional causal relationship between MDD and CT and SA using a Mendelian randomization (MR) design and performed exploratory analyses of MDD on CT and SA in different brain regions.

**Methods:**

Summary‐level data were obtained from two GWAS meta‐analysis studies: one screening for single nucleotide polymorphisms (SNPs) predicting the development of MDD (*n* = 135,458) and the other identifying SNPs predicting the magnitude of cortical thickness (CT) and surface area (SA) (*n* = 51,665).

**Results:**

The results showed that MDD caused a decrease in CT in the medial orbitofrontal region, a decrease in SA in the paracentral region, and an increase in SA in the lateral occipital region. C‐reactive protein, tumor necrosis factor alpha (TNF‐α), interleukin‐1β, and interleukin‐6 did not mediate the reduction. We also found that a reduction in CT in the precentral region and a reduction in SA in the orbitofrontal regions might be associated with a higher risk of MDD. Conclusion: Our study did not suggest an association between MDD and cortical CT and SA.

## Graphical abstract

Our study found that MDD caused a decrease in CT in the medial orbitofrontal region, a decrease in SA in the paracentral region, and an increase in SA in the lateral occipital region.

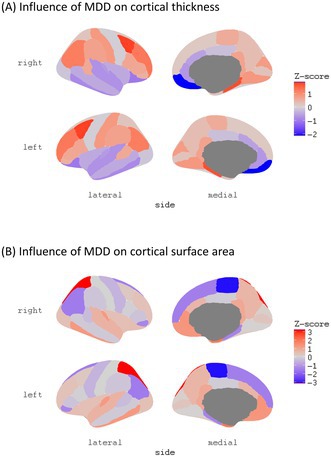

## 1 Introduction

Depression is a highly prevalent psychiatric disorder, and patients with depression manifest pessimistic thoughts about the future. Major depressive disorder (MDD) is characterized by persistent and recurrent depression, which makes it difficult to cure and prone to relapse. Therefore, physicians are keen to discover biomarkers that can predict the prognostic outcome of MDD and predictors of comorbidity after MDD.

A bidirectional association between MDD and abnormal brain structure has been noted and reported [1, 2]. MDD was found to be associated with a lower gray matter volume [2], suggesting a potential impact of MDD on brain structure and cognitive function, as low gray matter volume is an indicator of cognitive decline [3, 4, 5]. In addition to effects on gray matter volume, MDD has also been found to affect the cortical thickness (CT) and cortical surface area (SA) [6, 7, 8]. One study reporting on a cohort recruiting 2148 MDD patients and 7957 healthy controls showed that MDD was associated with reductions in SA in total and frontal regions [7]. Another study followed a group of MDD patients for 2 years and found that childhood maltreatment might lead to relapse of MDD, and that this detrimental effect might be mediated by abnormal cortical structure—mainly through the reduced SA in the right insula. If these findings were true and robust, they would shed light on the preventive treatment strategies for MDD by using neuromodulation modalities such as repetitive transcranial magnetic stimulation and neuroimaging biomarkers to predict MDD severity and relapse. However, traditional observational studies are prone to bias due to confounding factors and reverse causation, which challenges the robustness of the above findings. In addition, previous studies rarely evaluate the impact of MDD on differential brain regions, and vice versa.

The Mendelian randomization (MR) study design is increasingly being utilized to infer causal associations between two phenotypes. For example, the causal effect of serum lipid levels on the risk of cardiovascular disease was discovered using the MR design [9, 10], helping to translate lipid‐lowering drugs into cardiovascular disease prevention. The MR design uses genetic tools—usually single nucleotide polymorphisms (SNPs)—to infer the causal effect of an exposure on an outcome [11] that is less likely to be affected by confounding and reverse causation because the design is analogous to randomized controlled trials, and the genetic effect allele is randomly assigned at conception. Thus, we aimed to use the MR design to determine whether there is a bidirectional causal relationship between the risk of MDD and abnormal CT and SA and, if possible, to determine which brain regions are most affected.

## 2 Materials and Methods

### 2.1 Study Overview

**FIGURE 1:**
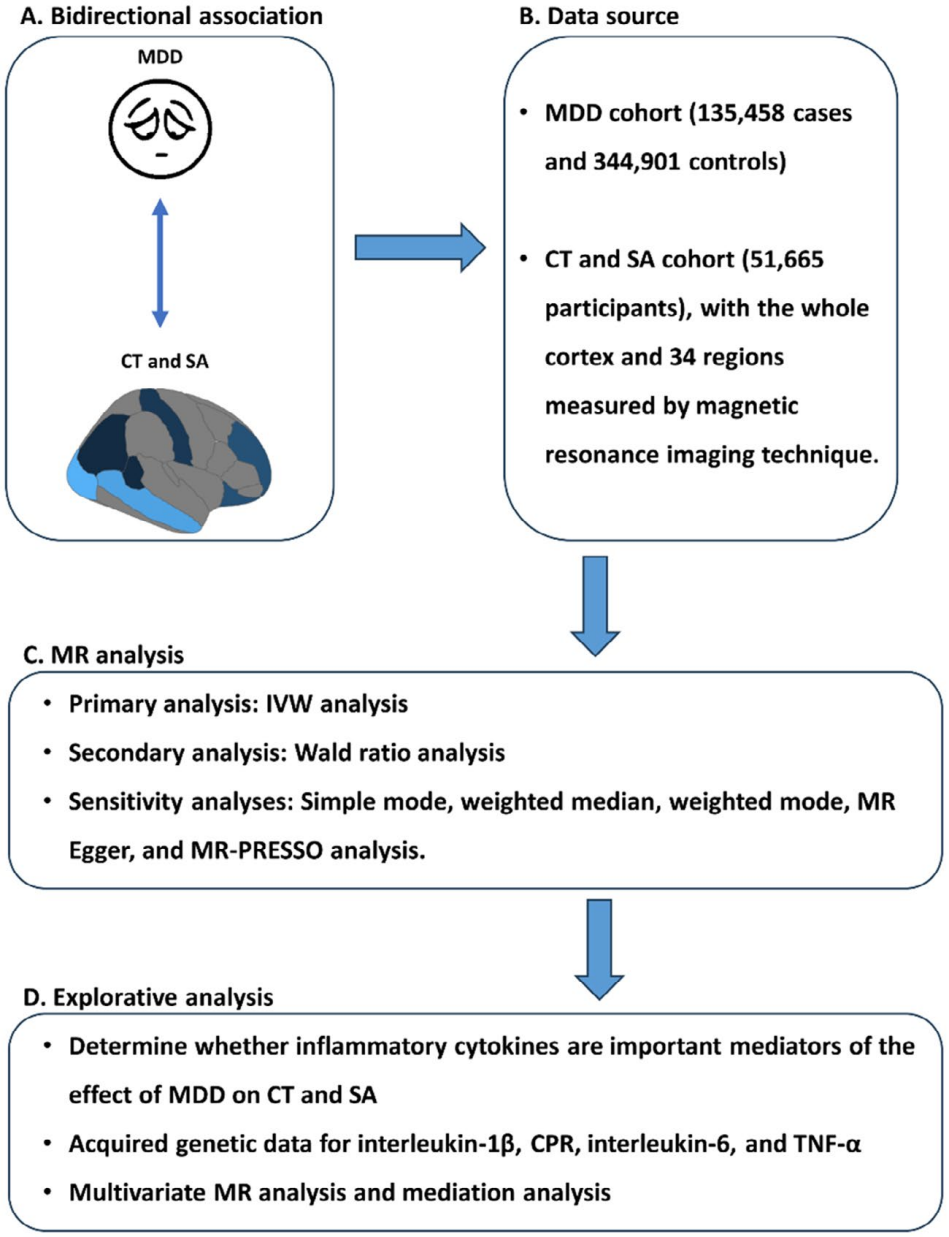
Study design. CRP, C‐reactive protein; CT, cortical thickness; IVW, inverse variance weighted; MDD, major depressive disorder; MR, Mendelian randomization; MR‐PRESSO, Mendelian Randomization Pleiotropy RESidual Sum and Outlier; SA, surface area; TNF‐α, tumor necrosis factor‐alpha.

We aimed to test two hypotheses: (1) MDD causes a reduction in CT and SA; (2) a reduction in CT and SA also increases the risk of MDD. We also aimed to clarify which brain regions are affected by MDD and which regions increase the risk of MDD. To test the hypotheses, we adopted the two‐sample MR design in which we acquired genetic instruments—in this study, we selected SNPs that predicted the risk of MDD—to infer the influence of MDD on CT and SA and vice versa. The study was conducted according to the guideline strengthening the reporting of observational studies in epidemiology using Mendelian randomization (STROBE‐MR) [12]. Ethical approval and informed consent were obtained at each participating center of the previously published GWAS studies [13, 14], and we used the summary‐level genetic data, and additional ethical approval was waived for this study. The study design is shown in Figure 1.

### 2.2 Data Source

Genetic data for MDD were obtained from a genome‐wide association meta‐analysis of 34 cohorts of European ancestry (135,458 MDD cases and 344,901 controls). Cases were required to meet international consensus criteria (DSM‐IV, ICD‐9, or ICD‐10) for MDD, and details of participant screening and characteristics have been described and reported elsewhere [15].

The genetic data, including genetic variants affecting cortical structure, were obtained from another genome‐wide association meta‐analysis of brain magnetic resonance imaging data from 51,665 participants of European ancestry [13]. In this meta‐analysis study, SA and CT of the entire cortex and 34 brain regions were analyzed, and the genetic variants predicting the size of SA and CT were determined.

### 2.3 Selection of Instruments

The selection of genetic instruments to infer the causal relationship between MDD and cortical structure is key to our study. The instruments should meet the three assumptions for MR design. First, the genetic instruments should be able to predict the risk of MDD or the size of CT and SA. In our study, SNPs were selected as the instruments, and the *p* value of the association between SNPs and MDD was set at 5e‐8. In examining the reverse causation of CT and SA on MDD, we established a *p* value threshold of 5e‐6. This decision was informed by the absence of significant SNPs for CT and SA in most brain regions under the more stringent threshold of 5e‐8, as well as the fact that even with more lenient thresholds (*p* < 1e‐6 and *p* < 1e‐5), only a single instrumental variable was identified for these exposures. To minimize the possibility of weak instrumental bias caused by the relaxation of the *p* value standard, we calculated the *F*‐statistic for the SNPs [16], and an *F*‐statistic > 10 would be justified as no risk of weak instrumental bias. The second assumption—the SNPs should not be confounded by potential confounders that could affect the risk of MDD or the magnitude of CT and SA—should also be met. In our study, the GWAS studies that provided the genetic data had adjusted for age and sex to minimize the impact of potential confounding issues; for example, older age was associated with thinner CT [17]. The third assumption—that the SNPs affect the size of CT and SA only through MDD—was ensured by clumping the genetic data using the most stringent criteria (*r* ^2^ < 0.001 and the clumping window within 10,000 kilobases) to control for linkage disequilibrium.

### 2.4 Data Analysis

The first hypothesis to be tested in this study was whether MDD has a causal effect on global CT and SA and which brain regions are significantly affected. The primary analysis used the inverse variance weighted (IVW) method. The SNPs that predicted the risk of MDD were extracted for their effect sizes (measured by *β* value) and corresponding standard errors on MDD, CT, and SA, respectively. The Wald ratio for each SNP was then calculated by dividing the *β* value of CT and SA by the *β* value of MDD, and the IVW analysis was performed to synthesize the Wald ratios and provide a general estimate of the effect of MDD on CT and SA. Five sensitivity analyses were performed to test the robustness of the IVW analysis: simple mode, weighted median, weighted mode, MR Egger, and Mendelian Randomization Pleiotropy RESidual Sum and Outlier (MR‐PRESSO) analysis. The benefits and mechanisms of sensitivity analyses have been described elsewhere [18]. In summary, the simple mode, weighted median, and weighted mode methods applied different rules to estimate the general effect of the included SNP, whereas MR Egger and MR‐PRESSO applied the regression method to estimate the pleiotropic effect and tested the third assumption of the MD design. Additionally, a leave‐one‐out analysis was conducted to ascertain whether a single SNP accounts for the majority of the causal effect. The Bonferroni method was employed to correct for the *p* value. Given that the primary objective was to examine the causal effect of MDD on SA and CT, as well as their reverse causal relationship, four comparisons were made. Therefore, the *p* value adjustment would be *α*(0.05)/4 = 0.0125. The remaining analyses were regarded as exploratory in nature. A total of 68 exploratory analyses were carried out to test the effect of MDD on CT and SA in differential brain regions. Subsequently, the *p* value adjustment for exploratory analyses would be *α*(0.05)/68 = 0.000735.

Because inflammation has been associated with the development and severity of depression [19] and might be a major contributor to the abnormal brain structure [20], we performed a mediation analysis by including serum levels of C‐reactive protein, interleukin‐1β, tumor necrosis factor‐alpha (TNF‐α), and interleukin‐6 in a multivariate MR model to examine whether these inflammatory cytokines mediate the effect of MDD on CT and SA. In the multivariate MR model, we incorporated serum concentrations of C‐reactive protein, interleukin‐1β, TNF‐α, and interleukin‐6 as exposures, alongside MDD status. The outcomes in the model were defined as alterations in brain structure. We subsequently estimated the effect sizes for each exposure on the specified outcomes. We also performed a two‐step MR mediation analysis to validate the results of the multivariate MR analysis, in which we first estimated the causal effect of MDD on inflammatory cytokines and secondly estimated the causal effect of inflammatory cytokines on CT and SA. The data sources and study population for these inflammatory cytokines have been reported elsewhere [21, 22, 23]. To minimize the risk of inflating *p* values due to multiple comparisons, we analyzed the brain regions significantly affected by MDD—defined as *p* < 0.05 in an IVW analysis.

For the second hypothesis—a reduction in CT or SA is associated with a higher risk of MDD—we repeated the MR analysis but treated CT and SA as exposure and the risk of MDD as the outcome. If only the SNP met the instrument selection standard, we performed the Wald ratio analysis only.

A power calculation was performed to ascertain the sufficiency of the sample size for the examination of the primary hypothesis. In accordance with the methodology of a previously published cohort study that investigated the correlation between cortical thickness and the pathogenesis of MDD [6], we assumed a type I error (*α*) of 0.05, a type II error (*β*) of 0.2, an anticipated causal effect of 0.004, and a correlation coefficient of 0.219 in the power calculation, which yielded a sample size of 8,959,908 [24].

To show the effect of MDD on CT and SA in a specific brain region, we calculated the *z*‐score by dividing the *β* provided in the IVW analysis by the corresponding standard error and presented the *z*‐scores in a brain map (presented according to the Desikan‐Killiany Atlas standard) to show the differential effect sizes of MDD. All analyses were performed using the R project software (version 4.2.2) and the TwoSampleMR package (version 0.5.6).

## 3 Results

### 3.1 The Selection of Instruments

For the analysis of the effect of MDD on CT and SA, 32 SNPs were selected for analysis. The mean *F*‐statistic of the instruments was 37, while the lowest *F*‐statistic was 30.4 (produced by SNP rs159963) and the highest was 79.9 (produced by SNP rs12552). For the analysis of the effect of CT and SA on MDD, most brain regions had only one SNP that met the screening standard, and these SNPs all had an *F*‐statistic above 10.

### 3.2 The Causal Effect of MDD on CT in Differential Regions

**FIGURE 2:**
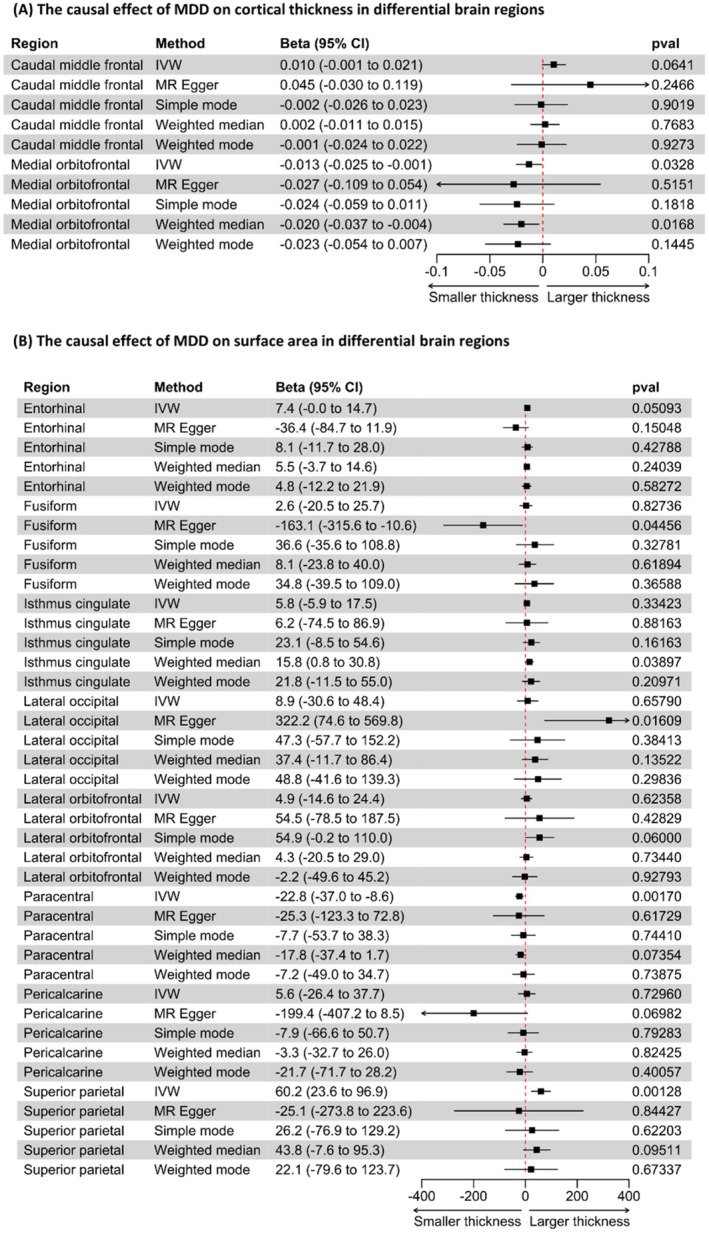
The causal effect of MDD on CT and SA in differential brain regions. (A, B) The effect size and corresponding 95% CI of MDD on CT and SA in differential brain regions, respectively. A negative beta value indicates that a higher risk of MDD is associated with a small size of CT or SA, and a positive value indicates the opposite. 95% CI, 95% confidence interval; CT, cortical thickness; IVW, inverse variance weighted; MDD, major depressive disorder; MR, Mendelian randomization; *p*val, *p* values; SA, surface area.

**FIGURE 3:**
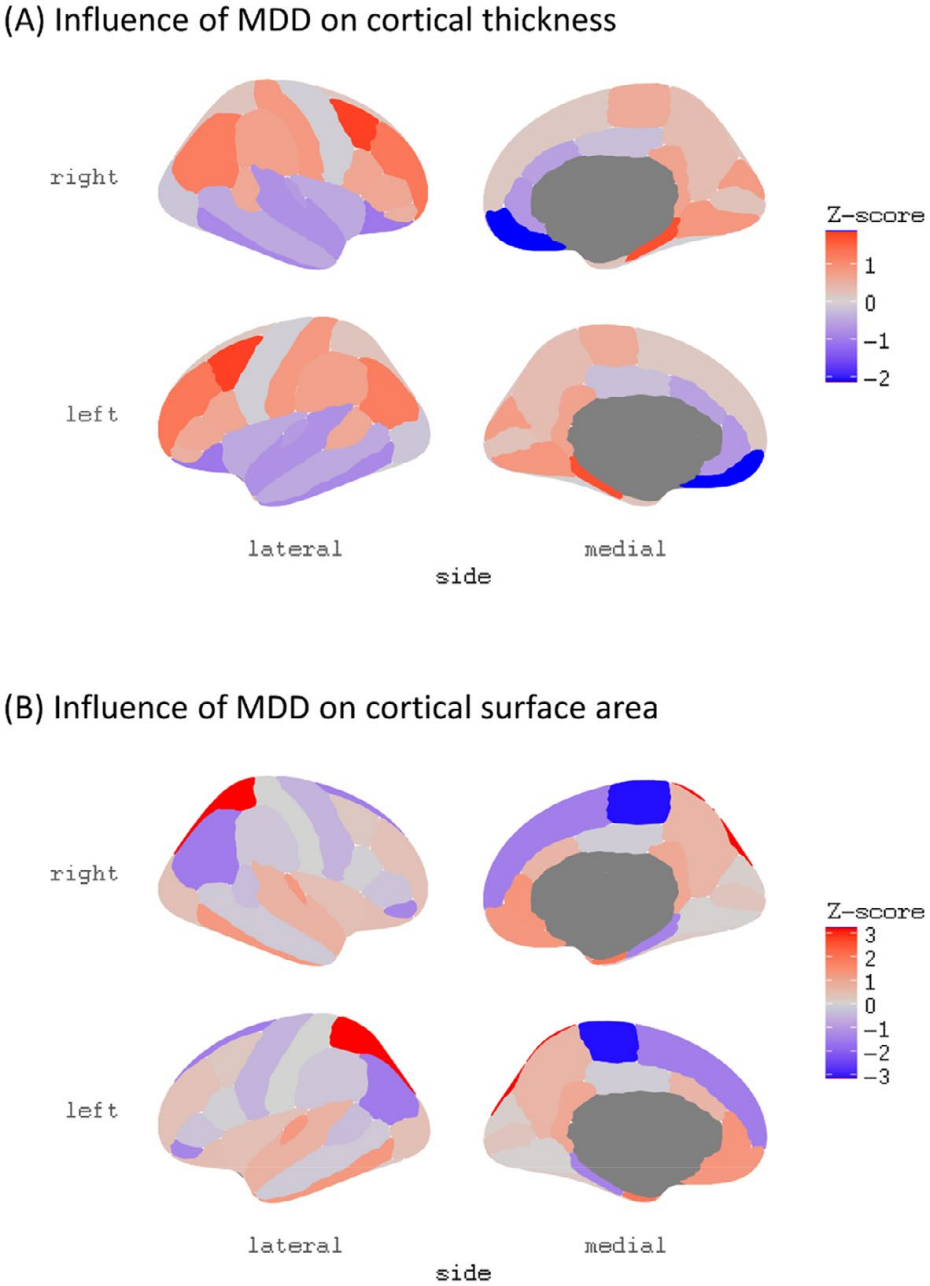
Influence of MDD on CT and SA measured by *z*‐score. (A, B) The influence of MDD on CT and SA, respectively, and the influence was measured using *z*‐scores. The *z*‐score was calculated by dividing the *β*‐value by its corresponding standard error, which was generated in the inverse variance weighted (IVW) analysis or the Wald ratio analysis. A negative *z*‐score value indicated that a higher risk of MDD was associated with a smaller size of CT or SA, and a positive value indicated the opposite. MDD, major depressive disorder.

The IVW analysis showed no effect of MDD on global CT but showed that MDD caused a reduction in the medial orbitofrontal region (beta −0.013, 95% CI −0.025 to −0.001, *p* = 0.033, Figure 2A). The weighted median analysis supported this finding (beta −0.02, 95% CI −0.037 to −0.004, *p* = 0.017, Figure 2A). The IVW analysis and other sensitivity analyses showed no evidence of a causal effect of MDD on CT in other brain regions (Table S1). The MR‐PRESSO analysis yielded a *p* value of 0.011 for the global test, prompting further investigation through an outlier test. This analysis revealed the absence of a significant effect attributable to an outlier. The leave‐one‐out analysis did not identify a single SNP that could explain the causal effect of MDD on global CT. However, the heterogeneity test yielded significant *p* values in the MR Egger and IVW models (MR Egger model, *Q* value 50.11, *p* value 0.012; IVW model, *Q* value 50.32, *p* value 0.016). However, further MR Egger regression analysis demonstrated the absence of a directional pleiotropic effect (MR Egger regression intercept, 0.0001; *p* value, 0.919). The results of the MR‐PRESSO, leave‐one‐out analysis, heterogeneity tests, and MR Egger regression analysis can be found in Table S2 and Figure S1. Figure 3A shows the effect of MDD on CT measured in different brain regions.

The multivariate and mediation analysis showed that CRP (*β* −0.01) and interleukin‐1β (−0.005) contributed to the reduction of CT in the medial orbitofrontal region (Table S3), but they did not mediate the effect of MDD, as the effect size did not decrease in the multivariate model (*β* −0.016). The two‐step MR mediation analysis showed that MDD had no causal effect on CRP, TNF‐α, interleukin‐1β, and interleukin‐6, nor did these inflammatory cytokines have effects on CT (Table S4).

### 3.3 The Causal Effect of MDD on SA in Differential Regions

IVW analysis showed no effect of MDD on global SA. However, the IVW analysis revealed that MDD caused a decrease in SA in the paracentral region (*β* −22.8, 95% CI −37 to −8.6, *p* = 0.002, Figure 2B) and an increase in SA in the superior parietal region (*β* 60.2, 95% CI 23.6 to 96.9, *p* = 0.001, Figure 2B). The IVW analysis showed no evidence of a causal effect of MDD on SA in other brain regions (Table S5), which was also confirmed in sensitivity analyses. The MR‐PRESSO analysis showed no significant *p* value in the global test (*p* value, 0.912). Leave‐one‐out analysis did not identify any single SNP that contributed significantly to the causal effect. Heterogeneity showed no significant difference (MR Egger model, *Q* value 39.97, *p* value 0.105; IVW model, *Q* value 40.86, *p* value 0.111), and MR Egger regression analysis showed no significant directional pleiotropic effect (MR Egger regression intercept, 122.83; *p* value, 0.447). The results of MR‐PRESSO, leave‐one‐out analysis, heterogeneity tests, and MR Egger regression analysis are available in Table S2 and Figure S1. Figure 3B shows the effect of MDD on CT measured in differential brain regions.

The multivariate and mediation analysis showed that interleukin‐6 (*β* −13.7) mediated part of the reduction of SA in the paracentral region, and the effect size of MDD decreased (*β* −15.9). The inflammatory cytokines did not mediate the effect of MDD on SA in the superior parietal region, as the effect size increased (*β* 77.8) (Table S3). The two‐step MR mediation analysis showed that MDD had no causal effect on CRP, TNF‐α, interleukin‐1β, and interleukin‐6, nor did these inflammatory cytokines have effects on SA (Table S4).

All the SNPs used for analysis are shown in Table S6.

### 3.4 The Causal Effect of CT on the Risk of MDD

**FIGURE 4:**
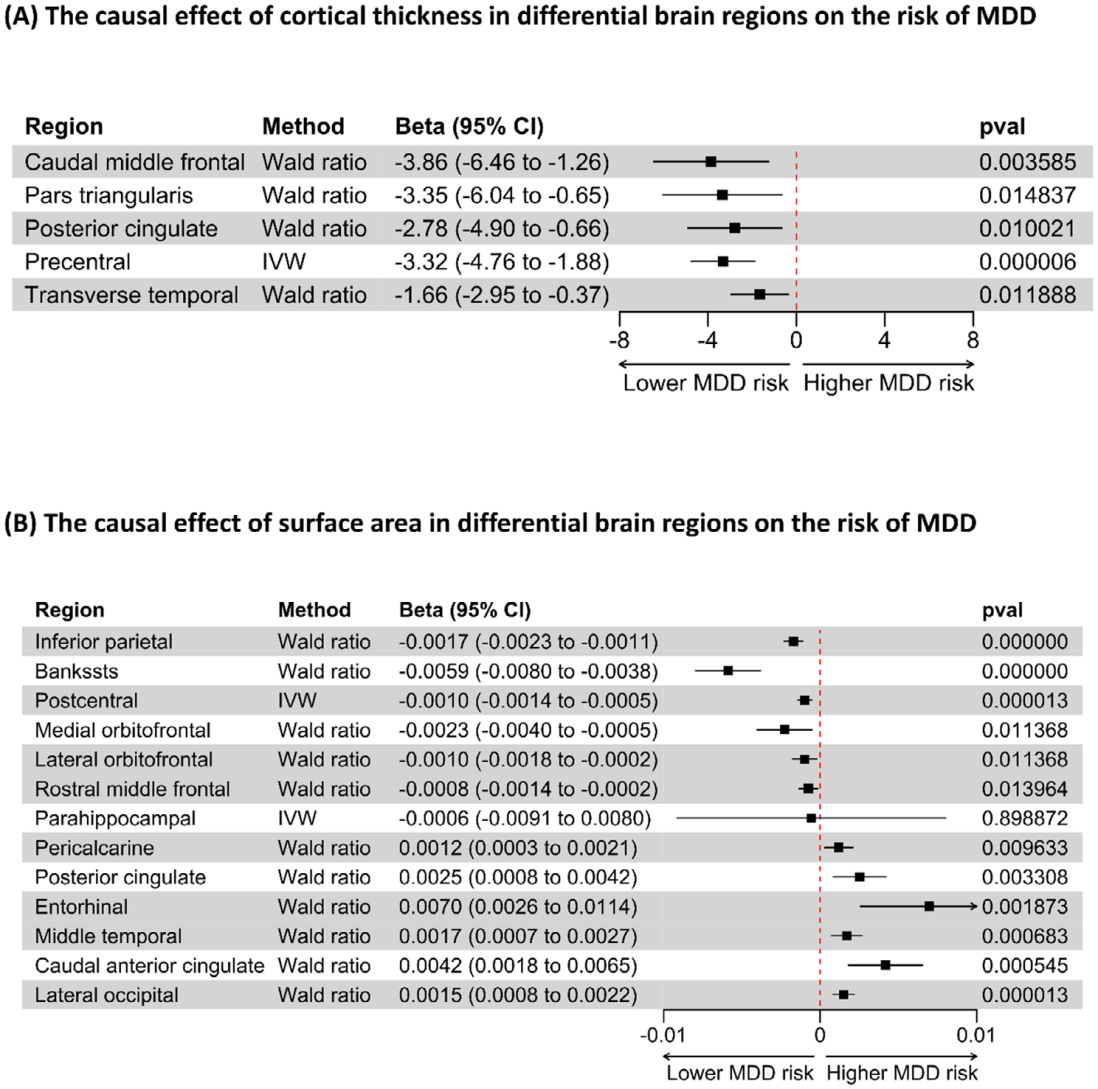
The causal effect of CT and SA in differential brain regions on the risk of MDD. (A, B) The effect of CT and SA, respectively, on the risk of MDD. 95% CI, 95% confidence interval; Bankssts, banks of the superior temporal sulcus; CT, cortical thickness; IVW, inverse variance weighted; MDD, major depressive disorder; *p*val, *p* values; SA, surface area.

**FIGURE 5:**
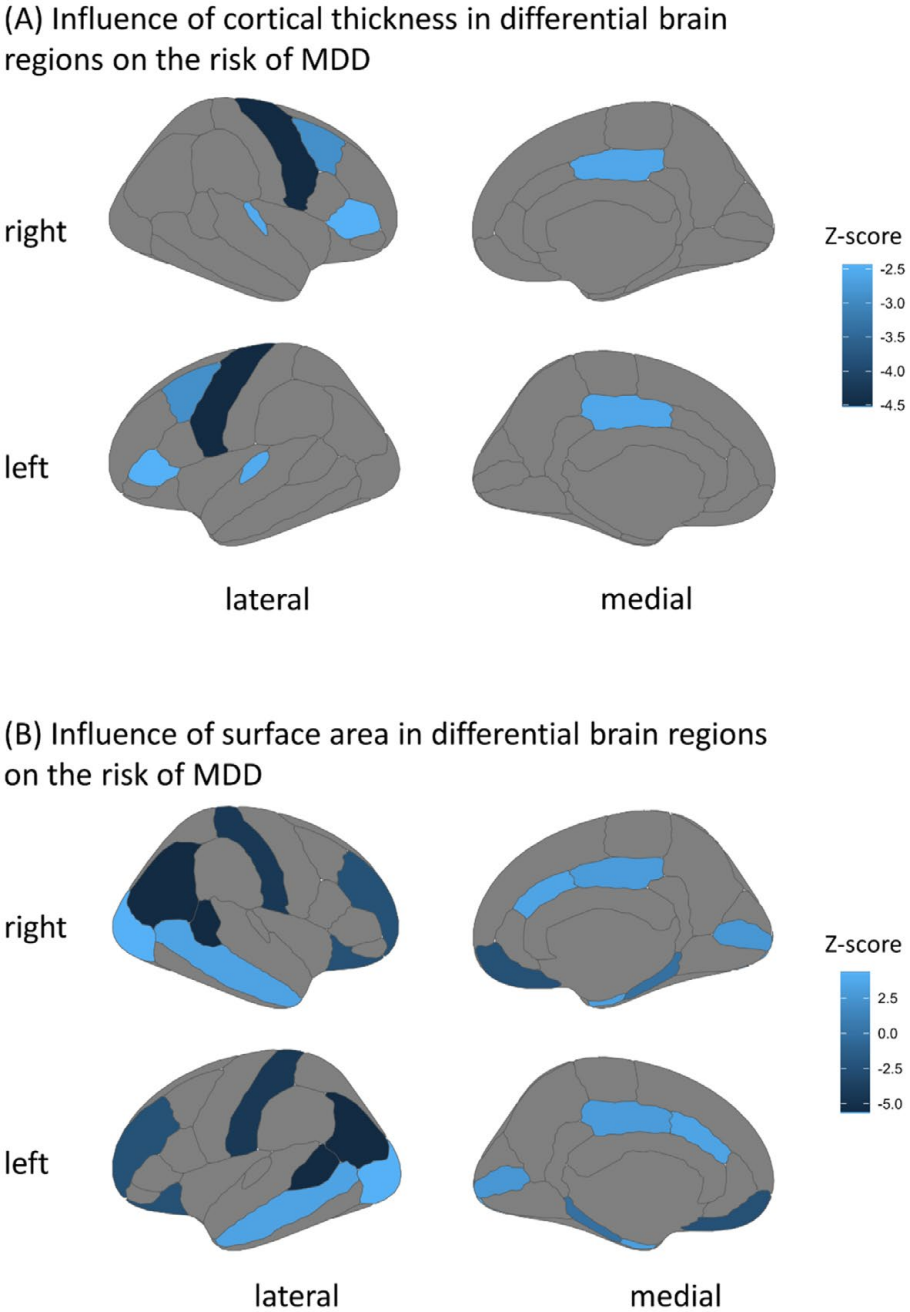
Influence of CT and SA in differential brain regions on the risk of MDD measured by *z*‐score. (A, B) The influence of CT and SA on the risk of MDD, respectively, and the influence was measured using *z*‐scores. The *z*‐score was calculated by dividing the *β*‐value by its corresponding standard error, which was generated in the inverse variance weighted (IVW) analysis or the Wald ratio analysis. MDD, major depressive disorder.

Due to the lack of sufficient genetic tools to predict CT size in brain regions, only five analyses were performed. One of the analyses used the IVW analysis, while the other four used the Wald ratio analysis, with only one SNP included. CT sizes in the caudal middle frontal, pars triangularis, posterior cingulate, precentral, and transverse temporal regions were associated with a higher risk of MDD (Figures 4A and 5A).

### 3.5 The Causal Effect of SA on the Risk of MDD

For the aforementioned reason, 13 analyses were conducted to test the causal effect of SA on the risk of MDD—two of them using the IVW method and the rest using the Wald ratio method. The analyses showed that smaller sizes of SA in the regions of the banks of the superior temporal sulcus, medial orbitofrontal, inferior parietal, lateral orbitofrontal, postcentral, rostral middle frontal, and parahippocampal were associated with a higher risk of MDD, whereas smaller SA sizes in the pericalcarine, lateral occipital, middle temporal, posterior cingulate, caudal anterior cingulate, and entorhinal regions were associated with a lower risk of MDD (Figures 4B and 5B).

Due to the limited number of SNPs available for assessing the impact of CT and SA on MDD—with only three analyses conducted using the IVW method, each incorporating just two SNPs—we did not proceed with MR‐Egger regression, MR‐PRESSO, or leave‐one‐out analyses because of an insufficient number of instrumental variables.

## 4 Discussion

Previous studies have reported an association between MDD and abnormal cortical structure. Our study further clarifies whether MDD causes a reduction in CT and SA, and whether CT and SA also have an impact on the pathogenesis of MDD. The study results found no evidence that MDD affected CT or SA in the entire cortex. However, we found that MDD was causally associated with a decrease in CT in the medial orbitofrontal region, a decrease in SA in the paracentral region, and an increase in SA in the lateral occipital region. Mediation analysis indicated that inflammatory cytokines did not mediate the effect of MDD on CT and SA. In the MR reverse causation analysis, CT reduction in the precentral region and SA reduction in the postcentral region were associated with a higher risk of MDD.

The strength of the study lies in the use of the MR design, which facilitates causal inference. Another strength is that we used the largest genome‐wide association study to date to ensure the statistical power of our causal inference because the MR study design tends to have lower statistical power than the traditional observational design [25]. In addition, we performed a mediation analysis and demonstrated the role of inflammatory cytokines in mediating the effect of MDD on CT and SA.

Structural and functional changes in the orbitofrontal regions in MDD patients have been observed in previous studies. The volume of the orbitofrontal cortex was found to be reduced in MDD patients in remission, and the medial orbitofrontal region was 32% smaller than in healthy controls [26]. Our study had similar and relevant findings, showing a reduction in CT in the medial orbitofrontal region and that a reduction in medial orbitofrontal SA was causally associated with a higher risk of MDD. These findings suggest a bidirectional relationship between MDD and CT and SA in the medial orbitofrontal region. One study showed that positive functional connectivity between the subgenual anterior cingulate cortex region and the medial orbitofrontal cortex could predict whether a patient would respond to repetitive transcranial magnetic stimulation (rTMS) at baseline [27], and the other study found that rTMS targeting the dorsolateral prefrontal cortex of MDD patients resulted in a significant increase in the orbitofrontal region [28].

Another important finding of our study was that the reductions in CT and/or SA in the precentral, paracentral, and postcentral regions were causally associated with a higher risk of MDD. Several published studies have reported similar results. For example, a reduction in CT in the paracentral and precentral cortex was found to be associated with anhedonia, one of the main symptoms of MDD [29]. In addition to structural abnormalities, abnormal brain function has also been reported in MDD patients. Disturbed regional homogeneity in the precentral and postcentral regions was found to be associated with melancholic MDD, a severe subtype of MDD [30]. A study using functional fMRI also found significantly different amplitudes of low‐frequency fluctuation (ALFF) values between MDD patients and healthy controls, and the difference was in the precentral and postcentral regions [31].

It is well established that inflammatory cytokines can influence neuronal function and cortical structure through a number of different mechanisms. Inflammatory cytokines modulate neurotransmitter systems, affect neuroplasticity, and even cross the blood–brain barrier to exert their effects directly on the central nervous system [32, 33, 34]. For example, cytokines such as interleukin‐1β, TNF‐α, and interleukin‐6, which can be synthesized in the central nervous system (CNS) or peripheral nervous system (PNS), have been demonstrated to influence neuronal activity and synaptic function [32]. A recent Mendelian randomization study showed a genetic association between major depressive disorder and reduced cortical gray matter volume [35], and the study suggested that interleukin‐6 may mediate the effect of MDD on gray matter volume. Furthermore, these inflammatory mediators can contribute to neuroinflammation, which has been implicated in the pathophysiology of various neurological and psychiatric disorders, including MDD [34]. In their review, Shinwan Kany et al. examine the role of cytokines such as interleukin‐1 in inflammatory processes, which can include effects on neuronal function and structure [33]. These cytokines, through their interaction with immune cells and neural tissues, have the potential to induce alterations in the brain's structure and function, which may contribute to the pathogenesis of psychiatric disorders such as depression. However, our study results did not support the hypothesis that the inflammatory cytokines mediated the effect of MDD on CT and SA. Future studies may focus on other factors that link the pathogenesis of MDD and the abnormal reduction of CT and SA, such as the severity of insomnia [36]. Insomnia severity was found to be associated with CT and SA shrinkage in MDD patients.

Our study had several limitations. First, despite the quasi‐randomized nature of the MR design, it might also be affected by confounding issues that were not investigated in this study. Second, the MR design has the disadvantage of low statistical power [25], so we might have missed some of the brain regions that are significantly affected by MDD. Third, we investigated the bidirectional relationship between the risk of MDD and changes in CT and SA; the results could only be generalized to the prevention of MDD. Whether the severity of MDD affects different brain regions or affects CT and SA differently is unknown, so the reduction in CT or SA could not be a biomarker for the detection of MDD severity, since our study did not support this conclusion.

## 5 Conclusions

Causal relationship between the risk of MDD and CT and SA was not confirmed in our study. The association between MDD and the medial orbitofrontal and paracentral regions should be further investigated. Inflammatory factors, CRP, TNF‐α, interleukin‐1β, and interleukin‐6 were unlikely mediators of the effect of MDD on cortical structure.

## Author Contributions

H.Z. and M.C. designed the study. Y.‐J.F., X.‐Y.W., S.‐J.F., and T.‐C.T. acquired the study data. M.C. and H.Z. analyzed and interpreted the data. H.Z. and Y.‐J.F. wrote the first draft of the manuscript. All authors revised the manuscript and approved it for publication.

## Disclosure

Role of the funder/sponsor: The sponsors had no role in the design and conduct of the study, and they had no role in the decision‐making process to submit the manuscript for publication.

Data access statement: All authors have full access to all data in the study and take responsibility for the integrity of the data and the accuracy of the data analysis, and all authors have the right to publish the data independently of any sponsor.

## Conflicts of Interest

The authors declare no conflicts of interest.

## Supporting information


**Table S1.** The causal effect of MDD on CT measured in differential brain regions.
**Table S2.** MR‐PRESSO, heterogeneity test, and MR Egger regression analysis.
**Table S3.** Multivariate Mendelian randomization and mediation analysis.
**Table S4.** Two‐step MR for the mediation analysis of MDD to SA and CT.
**Table S5.** The causal effect of MDD on SA measured in differential brain regions.
**Table S6.** SNPs included in the MR analysis.
**Figure S1.** Forest plots for leave one out analysis.

**Data S2.** Analysis code.

## Data Availability

Anonymized data not published in this article will be made available upon request to any qualified investigator, and the data will be available at pan.baidu.com after the article is published. The GWAS data of MDD and inflammatory cytokines (CRP, TNF‐α, interleukin‐1β, and interleukin‐6) were acquired from the IEU OpenGWAS project (https://gwas.mrcieu.ac.uk/), with access IDs ieu‐a‐1187, ieu‐b‐35, ebi‐a‐GCST004426, ebi‐a‐GCST004448, and ebi‐a‐GCST90012005. The GWAS data of cortical thickness and surface area were sourced from ENIGMA's website (https://enigma.ini.usc.edu/research/download‐enigma‐gwas‐results).
