# Supplementary material for: Causal Association Between Major Depressive Disorder and Cortical Structure: A Bidirectional Mendelian Randomization Study and Mediation Analysis
Source: CNS Neurosci Ther. 2025 Mar 9;31(3):e70319. doi: 10.1111/cns.70319 (PMC11890974; doi:10.1111/cns.70319)
Supplement: Supplementary file 1 — Table S1. The causal effect of MDD on CT measured in differential brain regions. Table S2. MR‐PRESSO, heterogeneity test, and MR Egger regression analysis. Table S3. Multivariate Mendelian randomization and mediation analysis. Table S4. Two‐step MR for the mediation analysis of MDD to SA and CT. Table S5. The causal effect of MDD on SA measured in differential brain regions. Table S6. SNPs included in the MR analysis. Figure S1. Forest plots for leave one out analysis. [file CNS-31-e70319-s002.docx]

**Supplementary Tables**

[Supplementary Table 1. The causal effect of MDD on CT measured in differential brain regions 1](#_Toc174042119)

[Supplementary Table 2. MR-PRESSO, heterogeneity test, and MR Egger regression analysis 1](#_Toc174042120)

[Supplementary Table 3. Multivariate Mendelian randomization and mediation analysis 1](#_Toc174042121)

[Supplementary Table 4. Two-step MR for the mediation analysis of MDD to SA and CT* 1](#_Toc174042122)

[Supplementary Table 5. The causal effect of MDD on SA measured in differential brain regions 1](#_Toc174042123)

[Supplementary Table 6. SNPs included in the MR analysis 1](#_Toc174042124)

[Supplementary Figure 1. Forest plots for leave one out analysis 1](#_Toc174042125)

# Supplementary Table 1. The causal effect of MDD on CT measured in differential brain regions

| **Region** | **Method** | **No. SNP** | **β** | **se** | **P-value** | **Z-score** |
| --- | --- | --- | --- | --- | --- | --- |
| bankssts | Inverse variance weighted | 32 | 0.005 | 0.007 | 0.533 | 0.624 |
| caudal anterior cingulate | Inverse variance weighted | 32 | -0.006 | 0.011 | 0.562 | -0.579 |
| caudal middle frontal | Inverse variance weighted | 32 | 0.010 | 0.006 | 0.064 | 1.852 |
| cuneus | Inverse variance weighted | 32 | 0.005 | 0.006 | 0.416 | 0.814 |
| entorhinal | Inverse variance weighted | 32 | 0.004 | 0.015 | 0.786 | 0.271 |
| frontal pole | Inverse variance weighted | 32 | -0.007 | 0.010 | 0.481 | -0.705 |
| fusiform | Inverse variance weighted | 32 | 0.000 | 0.005 | 0.974 | -0.032 |
| inferior parietal | Inverse variance weighted | 32 | 0.005 | 0.004 | 0.228 | 1.206 |
| inferior temporal | Inverse variance weighted | 32 | -0.005 | 0.006 | 0.388 | -0.863 |
| insula | Inverse variance weighted | 32 | -0.003 | 0.007 | 0.626 | -0.488 |
| isthmus cingulate | Inverse variance weighted | 32 | 0.006 | 0.009 | 0.480 | 0.707 |
| lateral occipital | Inverse variance weighted | 32 | -0.001 | 0.005 | 0.869 | -0.165 |
| lateral orbitofrontal | Inverse variance weighted | 32 | -0.007 | 0.006 | 0.293 | -1.052 |
| lingual | Inverse variance weighted | 32 | 0.004 | 0.005 | 0.379 | 0.880 |
| medial orbitofrontal | Inverse variance weighted | 32 | -0.013 | 0.006 | 0.033 | -2.135 |
| middle temporal | Inverse variance weighted | 32 | -0.002 | 0.005 | 0.628 | -0.484 |
| paracentral | Inverse variance weighted | 32 | 0.003 | 0.005 | 0.549 | 0.599 |
| parahippocampal | Inverse variance weighted | 32 | 0.023 | 0.013 | 0.084 | 1.727 |
| pars opercularis | Inverse variance weighted | 32 | 0.004 | 0.006 | 0.506 | 0.666 |
| pars orbitalis | Inverse variance weighted | 32 | 0.005 | 0.009 | 0.606 | 0.516 |
| pars triangularis | Inverse variance weighted | 32 | 0.003 | 0.005 | 0.519 | 0.645 |
| pericalcarine | Inverse variance weighted | 32 | 0.001 | 0.005 | 0.795 | 0.260 |
| postcentral | Inverse variance weighted | 32 | 0.004 | 0.005 | 0.393 | 0.854 |
| posterior cingulate | Inverse variance weighted | 32 | -0.002 | 0.007 | 0.816 | -0.232 |
| precentral | Inverse variance weighted | 32 | 0.000 | 0.004 | 0.955 | -0.057 |
| precuneus | Inverse variance weighted | 32 | 0.002 | 0.005 | 0.728 | 0.348 |
| rostral anterior cingulate | Inverse variance weighted | 32 | -0.008 | 0.011 | 0.486 | -0.697 |
| rostral middle frontal | Inverse variance weighted | 32 | 0.005 | 0.004 | 0.198 | 1.286 |
| superior frontal | Inverse variance weighted | 32 | 0.001 | 0.004 | 0.864 | 0.172 |
| superior parietal | Inverse variance weighted | 32 | 0.001 | 0.005 | 0.826 | 0.220 |
| superior temporal | Inverse variance weighted | 32 | -0.004 | 0.005 | 0.446 | -0.762 |
| supramarginal | Inverse variance weighted | 32 | 0.004 | 0.005 | 0.459 | 0.740 |
| temporal pole | Inverse variance weighted | 32 | 0.004 | 0.015 | 0.800 | 0.254 |
| transverse temporal | Inverse variance weighted | 32 | -0.006 | 0.009 | 0.511 | -0.657 |
| bankssts | MR Egger | 32 | -0.027 | 0.051 | 0.595 | -0.537 |
| caudal anterior cingulate | MR Egger | 32 | 0.085 | 0.074 | 0.264 | 1.139 |
| caudal middle frontal | MR Egger | 32 | 0.045 | 0.038 | 0.247 | 1.182 |
| cuneus | MR Egger | 32 | -0.015 | 0.043 | 0.734 | -0.343 |
| entorhinal | MR Egger | 32 | 0.059 | 0.104 | 0.577 | 0.565 |
| frontal pole | MR Egger | 32 | -0.013 | 0.069 | 0.852 | -0.188 |
| fusiform | MR Egger | 32 | -0.067 | 0.033 | 0.050 | -2.040 |
| inferior parietal | MR Egger | 32 | -0.016 | 0.030 | 0.601 | -0.529 |
| inferior temporal | MR Egger | 32 | 0.018 | 0.040 | 0.650 | 0.458 |
| insula | MR Egger | 32 | 0.042 | 0.047 | 0.371 | 0.908 |
| isthmus cingulate | MR Egger | 32 | -0.043 | 0.061 | 0.479 | -0.717 |
| lateral occipital | MR Egger | 32 | -0.048 | 0.032 | 0.144 | -1.502 |
| lateral orbitofrontal | MR Egger | 32 | -0.006 | 0.045 | 0.895 | -0.134 |
| lingual | MR Egger | 32 | 0.025 | 0.032 | 0.448 | 0.770 |
| medial orbitofrontal | MR Egger | 32 | -0.027 | 0.042 | 0.515 | -0.659 |
| middle temporal | MR Egger | 32 | -0.052 | 0.034 | 0.139 | -1.521 |
| paracentral | MR Egger | 32 | -0.017 | 0.036 | 0.650 | -0.459 |
| parahippocampal | MR Egger | 32 | -0.093 | 0.090 | 0.307 | -1.039 |
| pars opercularis | MR Egger | 32 | 0.009 | 0.040 | 0.824 | 0.224 |
| pars orbitalis | MR Egger | 32 | -0.035 | 0.064 | 0.585 | -0.552 |
| pars triangularis | MR Egger | 32 | 0.028 | 0.036 | 0.447 | 0.770 |
| pericalcarine | MR Egger | 32 | -0.062 | 0.035 | 0.084 | -1.789 |
| postcentral | MR Egger | 32 | 0.010 | 0.034 | 0.760 | 0.309 |
| posterior cingulate | MR Egger | 32 | 0.066 | 0.044 | 0.143 | 1.504 |
| precentral | MR Egger | 32 | 0.002 | 0.029 | 0.946 | 0.068 |
| precuneus | MR Egger | 32 | 0.002 | 0.032 | 0.942 | 0.073 |
| rostral anterior cingulate | MR Egger | 32 | 0.089 | 0.077 | 0.254 | 1.162 |
| rostral middle frontal | MR Egger | 32 | -0.013 | 0.027 | 0.627 | -0.491 |
| superior frontal | MR Egger | 32 | 0.021 | 0.030 | 0.498 | 0.685 |
| superior parietal | MR Egger | 32 | 0.011 | 0.037 | 0.764 | 0.303 |
| superior temporal | MR Egger | 32 | 0.020 | 0.034 | 0.557 | 0.593 |
| supramarginal | MR Egger | 32 | -0.017 | 0.033 | 0.605 | -0.523 |
| temporal pole | MR Egger | 32 | -0.071 | 0.104 | 0.501 | -0.681 |
| transverse temporal | MR Egger | 32 | 0.070 | 0.063 | 0.272 | 1.118 |
| bankssts | Simple mode | 32 | 0.011 | 0.018 | 0.558 | 0.592 |
| caudal anterior cingulate | Simple mode | 32 | -0.001 | 0.028 | 0.966 | -0.043 |
| caudal middle frontal | Simple mode | 32 | -0.002 | 0.013 | 0.902 | -0.124 |
| cuneus | Simple mode | 32 | 0.018 | 0.019 | 0.343 | 0.964 |
| entorhinal | Simple mode | 32 | -0.051 | 0.045 | 0.261 | -1.146 |
| frontal pole | Simple mode | 32 | -0.005 | 0.030 | 0.865 | -0.171 |
| fusiform | Simple mode | 32 | -0.009 | 0.016 | 0.579 | -0.561 |
| inferior parietal | Simple mode | 32 | 0.015 | 0.014 | 0.296 | 1.064 |
| inferior temporal | Simple mode | 32 | -0.023 | 0.016 | 0.180 | -1.372 |
| insula | Simple mode | 32 | 0.014 | 0.018 | 0.443 | 0.777 |
| isthmus cingulate | Simple mode | 32 | -0.010 | 0.022 | 0.666 | -0.436 |
| lateral occipital | Simple mode | 32 | -0.003 | 0.013 | 0.814 | -0.237 |
| lateral orbitofrontal | Simple mode | 32 | -0.012 | 0.017 | 0.483 | -0.710 |
| lingual | Simple mode | 32 | -0.003 | 0.013 | 0.799 | -0.257 |
| medial orbitofrontal | Simple mode | 32 | -0.024 | 0.018 | 0.182 | -1.366 |
| middle temporal | Simple mode | 32 | -0.007 | 0.014 | 0.647 | -0.462 |
| paracentral | Simple mode | 32 | 0.001 | 0.014 | 0.938 | 0.078 |
| parahippocampal | Simple mode | 32 | -0.019 | 0.036 | 0.595 | -0.536 |
| pars opercularis | Simple mode | 32 | 0.019 | 0.016 | 0.242 | 1.192 |
| pars orbitalis | Simple mode | 32 | 0.038 | 0.021 | 0.078 | 1.825 |
| pars triangularis | Simple mode | 32 | -0.007 | 0.014 | 0.593 | -0.540 |
| pericalcarine | Simple mode | 32 | -0.004 | 0.015 | 0.794 | -0.264 |
| postcentral | Simple mode | 32 | 0.012 | 0.011 | 0.279 | 1.103 |
| posterior cingulate | Simple mode | 32 | 0.005 | 0.017 | 0.776 | 0.287 |
| precentral | Simple mode | 32 | 0.006 | 0.012 | 0.613 | 0.511 |
| precuneus | Simple mode | 32 | 0.015 | 0.012 | 0.233 | 1.216 |
| rostral anterior cingulate | Simple mode | 32 | -0.029 | 0.028 | 0.323 | -1.004 |
| rostral middle frontal | Simple mode | 32 | 0.006 | 0.010 | 0.558 | 0.592 |
| superior frontal | Simple mode | 32 | -0.004 | 0.011 | 0.702 | -0.386 |
| superior parietal | Simple mode | 32 | -0.009 | 0.010 | 0.374 | -0.903 |
| superior temporal | Simple mode | 32 | -0.021 | 0.017 | 0.210 | -1.279 |
| supramarginal | Simple mode | 32 | -0.011 | 0.013 | 0.419 | -0.819 |
| temporal pole | Simple mode | 32 | 0.001 | 0.042 | 0.990 | 0.013 |
| transverse temporal | Simple mode | 32 | -0.027 | 0.025 | 0.292 | -1.072 |
| bankssts | Weighted median | 32 | 0.007 | 0.008 | 0.420 | 0.806 |
| caudal anterior cingulate | Weighted median | 32 | -0.002 | 0.013 | 0.900 | -0.126 |
| caudal middle frontal | Weighted median | 32 | 0.002 | 0.007 | 0.768 | 0.295 |
| cuneus | Weighted median | 32 | 0.004 | 0.008 | 0.644 | 0.463 |
| entorhinal | Weighted median | 32 | -0.021 | 0.020 | 0.298 | -1.041 |
| frontal pole | Weighted median | 32 | -0.007 | 0.014 | 0.627 | -0.486 |
| fusiform | Weighted median | 32 | -0.004 | 0.007 | 0.605 | -0.518 |
| inferior parietal | Weighted median | 32 | 0.007 | 0.005 | 0.188 | 1.317 |
| inferior temporal | Weighted median | 32 | -0.015 | 0.008 | 0.059 | -1.891 |
| insula | Weighted median | 32 | 0.003 | 0.008 | 0.722 | 0.356 |
| isthmus cingulate | Weighted median | 32 | 0.000 | 0.011 | 0.980 | 0.025 |
| lateral occipital | Weighted median | 32 | -0.003 | 0.006 | 0.655 | -0.447 |
| lateral orbitofrontal | Weighted median | 32 | -0.004 | 0.008 | 0.614 | -0.504 |
| lingual | Weighted median | 32 | 0.000 | 0.006 | 1.000 | 0.000 |
| medial orbitofrontal | Weighted median | 32 | -0.020 | 0.008 | 0.017 | -2.391 |
| middle temporal | Weighted median | 32 | -0.006 | 0.007 | 0.357 | -0.921 |
| paracentral | Weighted median | 32 | 0.003 | 0.007 | 0.667 | 0.430 |
| parahippocampal | Weighted median | 32 | -0.006 | 0.019 | 0.744 | -0.327 |
| pars opercularis | Weighted median | 32 | 0.007 | 0.007 | 0.267 | 1.110 |
| pars orbitalis | Weighted median | 32 | 0.021 | 0.011 | 0.047 | 1.989 |
| pars triangularis | Weighted median | 32 | -0.003 | 0.007 | 0.662 | -0.438 |
| pericalcarine | Weighted median | 32 | 0.000 | 0.007 | 0.989 | -0.014 |
| postcentral | Weighted median | 32 | 0.008 | 0.006 | 0.154 | 1.426 |
| posterior cingulate | Weighted median | 32 | 0.000 | 0.008 | 0.997 | 0.004 |
| precentral | Weighted median | 32 | -0.002 | 0.006 | 0.811 | -0.240 |
| precuneus | Weighted median | 32 | 0.005 | 0.005 | 0.311 | 1.012 |
| rostral anterior cingulate | Weighted median | 32 | -0.016 | 0.013 | 0.213 | -1.244 |
| rostral middle frontal | Weighted median | 32 | 0.005 | 0.005 | 0.342 | 0.951 |
| superior frontal | Weighted median | 32 | -0.001 | 0.006 | 0.910 | -0.113 |
| superior parietal | Weighted median | 32 | -0.007 | 0.006 | 0.238 | -1.181 |
| superior temporal | Weighted median | 32 | -0.007 | 0.008 | 0.360 | -0.916 |
| supramarginal | Weighted median | 32 | 0.003 | 0.006 | 0.546 | 0.603 |
| temporal pole | Weighted median | 32 | 0.003 | 0.018 | 0.865 | 0.170 |
| transverse temporal | Weighted median | 32 | -0.017 | 0.012 | 0.155 | -1.421 |
| bankssts | Weighted mode | 32 | 0.008 | 0.016 | 0.631 | 0.485 |
| caudal anterior cingulate | Weighted mode | 32 | -0.003 | 0.026 | 0.910 | -0.115 |
| caudal middle frontal | Weighted mode | 32 | -0.001 | 0.012 | 0.927 | -0.092 |
| cuneus | Weighted mode | 32 | -0.021 | 0.017 | 0.222 | -1.247 |
| entorhinal | Weighted mode | 32 | -0.051 | 0.043 | 0.244 | -1.187 |
| frontal pole | Weighted mode | 32 | -0.005 | 0.027 | 0.866 | -0.170 |
| fusiform | Weighted mode | 32 | -0.013 | 0.014 | 0.363 | -0.922 |
| inferior parietal | Weighted mode | 32 | 0.014 | 0.013 | 0.274 | 1.115 |
| inferior temporal | Weighted mode | 32 | -0.024 | 0.016 | 0.152 | -1.468 |
| insula | Weighted mode | 32 | 0.018 | 0.017 | 0.291 | 1.075 |
| isthmus cingulate | Weighted mode | 32 | -0.007 | 0.021 | 0.740 | -0.334 |
| lateral occipital | Weighted mode | 32 | -0.006 | 0.012 | 0.608 | -0.519 |
| lateral orbitofrontal | Weighted mode | 32 | 0.001 | 0.016 | 0.972 | 0.035 |
| lingual | Weighted mode | 32 | -0.002 | 0.011 | 0.850 | -0.191 |
| medial orbitofrontal | Weighted mode | 32 | -0.023 | 0.016 | 0.145 | -1.497 |
| middle temporal | Weighted mode | 32 | -0.006 | 0.014 | 0.646 | -0.464 |
| paracentral | Weighted mode | 32 | -0.008 | 0.014 | 0.554 | -0.598 |
| parahippocampal | Weighted mode | 32 | -0.021 | 0.033 | 0.525 | -0.642 |
| pars opercularis | Weighted mode | 32 | 0.021 | 0.015 | 0.161 | 1.436 |
| pars orbitalis | Weighted mode | 32 | 0.037 | 0.019 | 0.059 | 1.963 |
| pars triangularis | Weighted mode | 32 | -0.007 | 0.014 | 0.634 | -0.481 |
| pericalcarine | Weighted mode | 32 | -0.007 | 0.012 | 0.551 | -0.602 |
| postcentral | Weighted mode | 32 | 0.013 | 0.011 | 0.232 | 1.220 |
| posterior cingulate | Weighted mode | 32 | 0.003 | 0.015 | 0.831 | 0.215 |
| precentral | Weighted mode | 32 | -0.003 | 0.011 | 0.805 | -0.250 |
| precuneus | Weighted mode | 32 | 0.014 | 0.012 | 0.244 | 1.188 |
| rostral anterior cingulate | Weighted mode | 32 | -0.025 | 0.025 | 0.313 | -1.025 |
| rostral middle frontal | Weighted mode | 32 | 0.004 | 0.010 | 0.665 | 0.437 |
| superior frontal | Weighted mode | 32 | -0.007 | 0.011 | 0.551 | -0.602 |
| superior parietal | Weighted mode | 32 | -0.008 | 0.009 | 0.343 | -0.964 |
| superior temporal | Weighted mode | 32 | -0.020 | 0.017 | 0.244 | -1.189 |
| supramarginal | Weighted mode | 32 | -0.007 | 0.012 | 0.560 | -0.589 |
| temporal pole | Weighted mode | 32 | 0.002 | 0.036 | 0.963 | 0.047 |
| transverse temporal | Weighted mode | 32 | -0.024 | 0.027 | 0.375 | -0.900 |

Abbreviations: SNP, single nucleotide polymorphism. Se, standard error.

# Supplementary Table 2. MR-PRESSO, heterogeneity test, and MR Egger regression analysis

| **Tests** | **Global cortical thickness** | **Global surface area** |
| --- | --- | --- |
| **MR-PRESSO*** |  |  |
| Residual sum of squares | 53.42758 | 43.41169 |
| P-value | 0.011 | 0.128 |
| Outlier test | No significant outlier | No significant outlier |
| **Heterogeneity test**** |  |  |
| Q-value | 50.10706 | 39.9714 |
| P-value | 0.01208811 | 0.1054195 |
| **MR regression analysis**† |  |  |
| MR egger intercept | 0.000133339 | 122.826 |
| P-value | 0.9187717 | 0.4472086 |

**Abbreviations:** MR, mendelian randomization. MR-PRESSO, Mendelian Randomization Pleiotropy RESidual Sum and Outlier.

* The MR-PRESSO analysis showed no significant outlier for the MR analyses of MDD on cortical thickness and surface area.

** The heterogeneity test, employing the MR Egger regression approach, revealed significant variability in the MR analysis of cortical thickness, whereas no such heterogeneity was observed in the analysis of surface area.

† When the intercept of the MR Egger regression is near zero, it generally suggests that there is no significant pleiotropy bias present.

# Supplementary Table 3. Multivariate Mendelian randomization and mediation analysis

| **Exposure** | **Outcome** | **β** | **se** | **P-value** |
| --- | --- | --- | --- | --- |
| **Cortical thickness in medial orbitofrontal** |  |  |  |  |
|  | TNF-α | 0.006 | 0.012 | 0.644 |
|  | Interleukin-1β | -0.005 | 0.018 | 0.777 |
|  | Interleukin-6 | 0.013 | 0.019 | 0.491 |
|  | Major Depressive Disorder | -0.016 | 0.008 | 0.054 |
|  | C-Reactive protein | -0.010 | 0.039 | 0.798 |
| **Surface area in paracentral** |  |  |  |  |
|  | TNF-α | 9.265 | 17.025 | 0.586 |
|  | Interleukin-1β | 22.377 | 24.940 | 0.370 |
|  | Interleukin-6 | -13.695 | 26.044 | 0.599 |
|  | Major Depressive Disorder | -15.932 | 11.464 | 0.165 |
|  | C-Reactive protein | 63.446 | 53.582 | 0.236 |
| **Surface area in superior parietal** |  |  |  |  |
|  | TNF-α | 37.385 | 41.013 | 0.362 |
|  | Interleukin-1β | 9.979 | 60.056 | 0.868 |
|  | Interleukin-6 | -13.176 | 62.698 | 0.834 |
|  | Major Depressive Disorder | 77.847 | 27.601 | 0.005 |
|  | C-Reactive protein | -37.140 | 129.023 | 0.773 |

# Supplementary Table 4. Two-step MR for the mediation analysis of MDD to SA and CT*

| **Exposure** | **Outcome** | **β** | **se** | **P-value** |
| --- | --- | --- | --- | --- |
| **The first step** | | | | |
| MDD | C-Reactive protein | 0.05069876 | 0.04077363 | 0.2137129 |
| MDD | TNF-α | 0.03228835 | 0.1448517 | 0.8236084 |
| MDD | Interleukin-1β | -0.10547682 | 0.1119287 | 0.3460096 |
| MDD | Interleukin-6 | 0.07051101 | 0.07555332 | 0.3506850 |
| **The second step** | | | | |
| C-Reactive protein | Cortical thickness in medial orbitofrontal | 0.0018646304 | 0.002909312 | 0.5215759 |
| TNF-α |  | -0.005426816 | 0.003135314 | 0.0834752 |
| Interleukin-1β |  | -0.002154844 | 0.005186580 | 0.6778014 |
| Interleukin-6 |  | 0.00171723 | 0.005724098 | 0.7641772 |
| C-Reactive protein | Surface area in paracentral | -6.809532 | 3.838520 | 0.07606328 |
| TNF-α |  | 1.9152401 | 3.472983 | 0.5813127 |
| Interleukin-1β |  | -0.3639446 | 5.722256 | 0.9492875 |
| Interleukin-6 |  | -4.860332 | 6.554093 | 0.4583467 |
| C-Reactive protein | Surface area in superior parietal | 5.771869 | 9.767134 | 0.5545552 |
| TNF-α |  | 9.231856 | 14.43072 | 0.5223441 |
| Interleukin-1β |  | -16.69646 | 15.62860 | 0.2853729 |
| Interleukin-6 |  | -12.47338 | 18.02404 | 0.4889113 |

**Abbreviation:** MDD, major depressive disorder. TNF-α, tumor necrosis factor alpha.

* The two-step Mendelian randomization (MR) approach was employed to estimate the causal effect of major depressive disorder (MDD) on inflammatory cytokines and subsequently, the effect of these cytokines on the cortical thickness and surface area of brain regions affected by MDD, as indicated by the primary MR analysis. Negative β values were interpreted as indicating a negative correlation between exposure and outcome.

# Supplementary Table 5. The causal effect of MDD on SA measured in differential brain regions

| **Region** | **Method** | **No. SNP** | **β** | **se** | **P-value** | **Z-score** |
| --- | --- | --- | --- | --- | --- | --- |
| bankssts | Inverse variance weighted | 32 | -2.401 | 6.057 | 0.692 | -0.396 |
| bankssts | MR Egger | 32 | -24.345 | 41.532 | 0.562 | -0.586 |
| bankssts | Simple mode | 32 | -12.933 | 19.561 | 0.513 | -0.661 |
| bankssts | Weighted median | 32 | -2.876 | 7.948 | 0.717 | -0.362 |
| bankssts | Weighted mode | 32 | -15.910 | 18.129 | 0.387 | -0.878 |
| caudal anterior cingulate | Inverse variance weighted | 32 | 4.270 | 6.394 | 0.504 | 0.668 |
| caudal anterior cingulate | MR Egger | 32 | 48.748 | 43.310 | 0.269 | 1.126 |
| caudal anterior cingulate | Simple mode | 32 | 4.851 | 16.702 | 0.773 | 0.290 |
| caudal anterior cingulate | Weighted median | 32 | 7.403 | 7.289 | 0.310 | 1.016 |
| caudal anterior cingulate | Weighted mode | 32 | 5.841 | 14.997 | 0.700 | 0.389 |
| caudal middle frontal | Inverse variance weighted | 32 | 3.509 | 12.547 | 0.780 | 0.280 |
| caudal middle frontal | MR Egger | 32 | 31.177 | 86.309 | 0.720 | 0.361 |
| caudal middle frontal | Simple mode | 32 | 18.570 | 34.710 | 0.596 | 0.535 |
| caudal middle frontal | Weighted median | 32 | 8.811 | 16.773 | 0.599 | 0.525 |
| caudal middle frontal | Weighted mode | 32 | 13.935 | 32.347 | 0.670 | 0.431 |
| cuneus | Inverse variance weighted | 32 | 1.168 | 9.329 | 0.900 | 0.125 |
| cuneus | MR Egger | 32 | -59.856 | 63.330 | 0.352 | -0.945 |
| cuneus | Simple mode | 32 | -33.573 | 25.977 | 0.206 | -1.292 |
| cuneus | Weighted median | 32 | -8.425 | 11.094 | 0.448 | -0.759 |
| cuneus | Weighted mode | 32 | -28.770 | 22.960 | 0.220 | -1.253 |
| entorhinal | MR Egger | 32 | -36.392 | 24.663 | 0.150 | -1.476 |
| entorhinal | Simple mode | 32 | 8.134 | 10.125 | 0.428 | 0.803 |
| entorhinal | Weighted median | 32 | 5.484 | 4.671 | 0.240 | 1.174 |
| entorhinal | Weighted mode | 32 | 4.827 | 8.693 | 0.583 | 0.555 |
| frontal pole | Inverse variance weighted | 32 | -0.297 | 1.361 | 0.827 | -0.218 |
| frontal pole | MR Egger | 32 | 8.556 | 9.225 | 0.361 | 0.928 |
| frontal pole | Simple mode | 32 | -0.676 | 3.873 | 0.862 | -0.175 |
| frontal pole | Weighted median | 32 | -0.524 | 1.890 | 0.782 | -0.277 |
| frontal pole | Weighted mode | 32 | -0.578 | 3.563 | 0.872 | -0.162 |
| fusiform | Inverse variance weighted | 32 | 2.572 | 11.792 | 0.827 | 0.218 |
| fusiform | Simple mode | 32 | 36.626 | 36.839 | 0.328 | 0.994 |
| fusiform | Weighted median | 32 | 8.105 | 16.297 | 0.619 | 0.497 |
| fusiform | Weighted mode | 32 | 34.768 | 37.887 | 0.366 | 0.918 |
| inferior parietal | Inverse variance weighted | 32 | -31.204 | 20.298 | 0.124 | -1.537 |
| inferior parietal | MR Egger | 32 | 176.800 | 134.510 | 0.199 | 1.314 |
| inferior parietal | Simple mode | 32 | -0.076 | 56.924 | 0.999 | -0.001 |
| inferior parietal | Weighted median | 32 | -9.745 | 28.362 | 0.731 | -0.344 |
| inferior parietal | Weighted mode | 32 | -0.076 | 53.546 | 0.999 | -0.001 |
| inferior temporal | Inverse variance weighted | 32 | 17.972 | 13.953 | 0.198 | 1.288 |
| inferior temporal | MR Egger | 32 | 139.112 | 93.495 | 0.147 | 1.488 |
| inferior temporal | Simple mode | 32 | -30.390 | 41.821 | 0.473 | -0.727 |
| inferior temporal | Weighted median | 32 | -8.774 | 18.708 | 0.639 | -0.469 |
| inferior temporal | Weighted mode | 32 | -23.881 | 36.398 | 0.517 | -0.656 |
| insula | Inverse variance weighted | 32 | 4.233 | 9.187 | 0.645 | 0.461 |
| insula | MR Egger | 32 | -75.575 | 61.563 | 0.229 | -1.228 |
| insula | Simple mode | 32 | -10.617 | 21.172 | 0.620 | -0.501 |
| insula | Weighted median | 32 | -7.283 | 10.946 | 0.506 | -0.665 |
| insula | Weighted mode | 32 | -13.544 | 21.660 | 0.536 | -0.625 |
| isthmus cingulate | Inverse variance weighted | 32 | 5.772 | 5.977 | 0.334 | 0.966 |
| isthmus cingulate | MR Egger | 32 | 6.183 | 41.171 | 0.882 | 0.150 |
| isthmus cingulate | Simple mode | 32 | 23.074 | 16.093 | 0.162 | 1.434 |
| isthmus cingulate | Weighted mode | 32 | 21.753 | 16.982 | 0.210 | 1.281 |
| lateral occipital | Inverse variance weighted | 32 | 8.925 | 20.154 | 0.658 | 0.443 |
| lateral occipital | Simple mode | 32 | 47.273 | 53.547 | 0.384 | 0.883 |
| lateral occipital | Weighted median | 32 | 37.367 | 25.015 | 0.135 | 1.494 |
| lateral occipital | Weighted mode | 32 | 48.813 | 46.149 | 0.298 | 1.058 |
| lateral orbitofrontal | Inverse variance weighted | 32 | 4.877 | 9.937 | 0.624 | 0.491 |
| lateral orbitofrontal | MR Egger | 32 | 54.495 | 67.864 | 0.428 | 0.803 |
| lateral orbitofrontal | Weighted median | 32 | 4.281 | 12.619 | 0.734 | 0.339 |
| lateral orbitofrontal | Weighted mode | 32 | -2.204 | 24.173 | 0.928 | -0.091 |
| lingual | Inverse variance weighted | 32 | 0.715 | 18.099 | 0.968 | 0.039 |
| lingual | Simple mode | 32 | -16.666 | 42.829 | 0.700 | -0.389 |
| lingual | Weighted median | 32 | -13.830 | 20.938 | 0.509 | -0.661 |
| lingual | Weighted mode | 32 | -16.666 | 41.512 | 0.691 | -0.401 |
| medial orbitofrontal | Inverse variance weighted | 32 | 10.123 | 7.656 | 0.186 | 1.322 |
| medial orbitofrontal | MR Egger | 32 | 12.347 | 52.775 | 0.817 | 0.234 |
| medial orbitofrontal | Simple mode | 32 | -2.398 | 18.798 | 0.899 | -0.128 |
| medial orbitofrontal | Weighted median | 32 | 3.843 | 9.620 | 0.690 | 0.399 |
| medial orbitofrontal | Weighted mode | 32 | -0.779 | 19.241 | 0.968 | -0.040 |
| middle temporal | Inverse variance weighted | 32 | -1.906 | 11.232 | 0.865 | -0.170 |
| middle temporal | MR Egger | 32 | 54.730 | 76.178 | 0.478 | 0.718 |
| middle temporal | Simple mode | 32 | -20.437 | 33.497 | 0.546 | -0.610 |
| middle temporal | Weighted median | 32 | -11.561 | 16.449 | 0.482 | -0.703 |
| middle temporal | Weighted mode | 32 | -21.946 | 28.210 | 0.442 | -0.778 |
| paracentral | MR Egger | 32 | -25.263 | 50.031 | 0.617 | -0.505 |
| paracentral | Simple mode | 32 | -7.730 | 23.470 | 0.744 | -0.329 |
| paracentral | Weighted mode | 32 | -7.185 | 21.352 | 0.739 | -0.337 |
| parahippocampal | Inverse variance weighted | 32 | -4.903 | 3.519 | 0.164 | -1.393 |
| parahippocampal | Simple mode | 32 | -11.141 | 10.038 | 0.276 | -1.110 |
| parahippocampal | Weighted mode | 32 | -14.500 | 9.890 | 0.153 | -1.466 |
| pars opercularis | Inverse variance weighted | 32 | -0.578 | 9.496 | 0.952 | -0.061 |
| pars opercularis | MR Egger | 32 | 25.634 | 65.264 | 0.697 | 0.393 |
| pars opercularis | Simple mode | 32 | 8.625 | 26.005 | 0.742 | 0.332 |
| pars opercularis | Weighted median | 32 | 5.682 | 12.031 | 0.637 | 0.472 |
| pars opercularis | Weighted mode | 32 | 10.118 | 22.999 | 0.663 | 0.440 |
| pars orbitalis | Inverse variance weighted | 32 | -3.824 | 2.832 | 0.177 | -1.350 |
| pars orbitalis | MR Egger | 32 | 18.584 | 19.196 | 0.341 | 0.968 |
| pars orbitalis | Simple mode | 32 | 1.126 | 8.480 | 0.895 | 0.133 |
| pars orbitalis | Weighted median | 32 | -3.035 | 4.121 | 0.461 | -0.736 |
| pars orbitalis | Weighted mode | 32 | -0.039 | 7.217 | 0.996 | -0.005 |
| pars triangularis | Inverse variance weighted | 32 | -2.038 | 7.483 | 0.785 | -0.272 |
| pars triangularis | MR Egger | 32 | -18.122 | 50.710 | 0.723 | -0.357 |
| pars triangularis | Simple mode | 32 | -13.235 | 18.797 | 0.487 | -0.704 |
| pars triangularis | Weighted median | 32 | -3.875 | 10.081 | 0.701 | -0.384 |
| pars triangularis | Weighted mode | 32 | -11.806 | 20.810 | 0.575 | -0.567 |
| pericalcarine | Inverse variance weighted | 32 | 5.646 | 16.335 | 0.730 | 0.346 |
| pericalcarine | Simple mode | 32 | -7.926 | 29.919 | 0.793 | -0.265 |
| pericalcarine | Weighted median | 32 | -3.326 | 14.977 | 0.824 | -0.222 |
| pericalcarine | Weighted mode | 32 | -21.726 | 25.490 | 0.401 | -0.852 |
| postcentral | Inverse variance weighted | 32 | 0.147 | 14.614 | 0.992 | 0.010 |
| postcentral | MR Egger | 32 | -112.698 | 98.621 | 0.262 | -1.143 |
| postcentral | Simple mode | 32 | -33.780 | 39.808 | 0.403 | -0.849 |
| postcentral | Weighted median | 32 | -4.059 | 19.282 | 0.833 | -0.210 |
| postcentral | Weighted mode | 32 | -27.795 | 40.200 | 0.494 | -0.691 |
| posterior cingulate | Inverse variance weighted | 32 | -0.900 | 7.322 | 0.902 | -0.123 |
| posterior cingulate | MR Egger | 32 | 9.067 | 50.437 | 0.859 | 0.180 |
| posterior cingulate | Simple mode | 32 | -7.567 | 22.833 | 0.743 | -0.331 |
| posterior cingulate | Weighted median | 32 | 0.038 | 8.330 | 0.996 | 0.005 |
| posterior cingulate | Weighted mode | 32 | -11.060 | 22.381 | 0.625 | -0.494 |
| precentral | Inverse variance weighted | 32 | -8.429 | 15.330 | 0.582 | -0.550 |
| precentral | MR Egger | 32 | -11.265 | 104.311 | 0.915 | -0.108 |
| precentral | Simple mode | 32 | -36.104 | 43.889 | 0.417 | -0.823 |
| precentral | Weighted median | 32 | -23.987 | 21.249 | 0.259 | -1.129 |
| precentral | Weighted mode | 32 | -34.068 | 45.460 | 0.459 | -0.749 |
| precuneus | Inverse variance weighted | 32 | 8.987 | 13.094 | 0.492 | 0.686 |
| precuneus | MR Egger | 32 | -118.489 | 88.827 | 0.192 | -1.334 |
| precuneus | Simple mode | 32 | -2.514 | 35.989 | 0.945 | -0.070 |
| precuneus | Weighted median | 32 | -1.364 | 18.760 | 0.942 | -0.073 |
| precuneus | Weighted mode | 32 | -3.568 | 31.868 | 0.912 | -0.112 |
| rostral anterior cingulate | Inverse variance weighted | 32 | 7.292 | 5.330 | 0.171 | 1.368 |
| rostral anterior cingulate | MR Egger | 32 | -7.949 | 36.636 | 0.830 | -0.217 |
| rostral anterior cingulate | Simple mode | 32 | 5.179 | 16.969 | 0.762 | 0.305 |
| rostral anterior cingulate | Weighted median | 32 | 9.570 | 6.824 | 0.161 | 1.402 |
| rostral anterior cingulate | Weighted mode | 32 | 8.589 | 14.304 | 0.553 | 0.600 |
| rostral middle frontal | Inverse variance weighted | 32 | 9.251 | 22.322 | 0.679 | 0.414 |
| rostral middle frontal | MR Egger | 32 | 144.513 | 151.858 | 0.349 | 0.952 |
| rostral middle frontal | Simple mode | 32 | 0.352 | 54.469 | 0.995 | 0.006 |
| rostral middle frontal | Weighted median | 32 | 8.254 | 25.730 | 0.748 | 0.321 |
| rostral middle frontal | Weighted mode | 32 | 2.237 | 53.416 | 0.967 | 0.042 |
| superior frontal | Inverse variance weighted | 32 | -31.393 | 21.148 | 0.138 | -1.484 |
| superior frontal | Simple mode | 32 | -52.162 | 61.625 | 0.404 | -0.846 |
| superior frontal | Weighted median | 32 | -34.562 | 28.101 | 0.219 | -1.230 |
| superior frontal | Weighted mode | 32 | -37.708 | 64.286 | 0.562 | -0.587 |
| superior parietal | MR Egger | 32 | -25.141 | 126.885 | 0.844 | -0.198 |
| superior parietal | Simple mode | 32 | 26.177 | 52.569 | 0.622 | 0.498 |
| superior parietal | Weighted mode | 32 | 22.063 | 51.844 | 0.673 | 0.426 |
| superior temporal | Inverse variance weighted | 32 | 10.020 | 13.465 | 0.457 | 0.744 |
| superior temporal | MR Egger | 32 | -114.101 | 89.865 | 0.214 | -1.270 |
| superior temporal | Simple mode | 32 | -52.756 | 46.701 | 0.267 | -1.130 |
| superior temporal | Weighted median | 32 | 17.309 | 17.161 | 0.313 | 1.009 |
| superior temporal | Weighted mode | 32 | -54.575 | 44.568 | 0.230 | -1.225 |
| supramarginal | Inverse variance weighted | 32 | -3.608 | 15.007 | 0.810 | -0.240 |
| supramarginal | MR Egger | 32 | -23.030 | 103.202 | 0.825 | -0.223 |
| supramarginal | Simple mode | 32 | 30.830 | 45.553 | 0.504 | 0.677 |
| supramarginal | Weighted median | 32 | 4.897 | 22.205 | 0.825 | 0.221 |
| supramarginal | Weighted mode | 32 | 16.864 | 43.918 | 0.704 | 0.384 |
| temporal pole | Inverse variance weighted | 32 | 3.168 | 2.649 | 0.232 | 1.196 |
| temporal pole | MR Egger | 32 | -5.792 | 18.180 | 0.752 | -0.319 |
| temporal pole | Simple mode | 32 | 4.132 | 7.352 | 0.578 | 0.562 |
| temporal pole | Weighted median | 32 | 2.730 | 3.261 | 0.403 | 0.837 |
| temporal pole | Weighted mode | 32 | 4.757 | 6.435 | 0.465 | 0.739 |
| transverse temporal | Inverse variance weighted | 32 | 2.986 | 2.336 | 0.201 | 1.279 |
| transverse temporal | MR Egger | 32 | 13.768 | 15.834 | 0.391 | 0.870 |
| transverse temporal | Simple mode | 32 | 5.767 | 7.191 | 0.429 | 0.802 |
| transverse temporal | Weighted median | 32 | 2.763 | 3.440 | 0.422 | 0.803 |
| transverse temporal | Weighted mode | 32 | 4.391 | 6.862 | 0.527 | 0.640 |
| entorhinal | Inverse variance weighted | 32 | 7.353 | 3.767 | 0.051 | 1.952 |
| fusiform | MR Egger | 32 | -163.105 | 77.794 | 0.045 | -2.097 |
| isthmus cingulate | Weighted median | 32 | 15.794 | 7.650 | 0.039 | 2.064 |
| lateral occipital | MR Egger | 32 | 322.232 | 126.326 | 0.016 | 2.551 |
| lateral orbitofrontal | Simple mode | 32 | 54.907 | 28.126 | 0.060 | 1.952 |
| lingual | MR Egger | 32 | -209.177 | 118.541 | 0.088 | -1.765 |
| paracentral | Inverse variance weighted | 32 | -22.783 | 7.261 | 0.002 | -3.138 |
| paracentral | Weighted median | 32 | -17.840 | 9.969 | 0.074 | -1.789 |
| parahippocampal | MR Egger | 32 | -43.414 | 23.851 | 0.079 | -1.820 |
| parahippocampal | Weighted median | 32 | -8.440 | 5.062 | 0.095 | -1.667 |
| pericalcarine | MR Egger | 32 | -199.356 | 106.030 | 0.070 | -1.880 |
| superior frontal | MR Egger | 32 | 256.042 | 135.740 | 0.069 | 1.886 |
| superior parietal | Inverse variance weighted | 32 | 60.226 | 18.706 | 0.001 | 3.220 |
| superior parietal | Weighted median | 32 | 43.847 | 26.271 | 0.095 | 1.669 |

# Supplementary Table 6. SNPs included in the MR analysis

| **SNP** | **Effect allele** | **Other allele** | **Beta** | **se** | **P value** |
| --- | --- | --- | --- | --- | --- |
| ***SNPs that are closely related to MDD*** | | | | | |
| rs10149470 | G | A | 0.0289964 | 0.0049 | 3.05401E-09 |
| rs10950398 | A | G | 0.0274984 | 0.0049 | 2.548E-08 |
| rs10959913 | G | T | -0.0333961 | 0.0057 | 5.06198E-09 |
| rs11135349 | C | A | 0.0293979 | 0.0048 | 1.09199E-09 |
| rs11643192 | A | C | 0.0270021 | 0.0049 | 3.359E-08 |
| rs11663393 | A | G | 0.0278 | 0.0049 | 1.64502E-08 |
| rs11682175 | C | T | 0.0281012 | 0.0048 | 4.68004E-09 |
| rs1226412 | T | C | 0.0332026 | 0.0059 | 2.38402E-08 |
| rs12552 | G | A | -0.0428966 | 0.0048 | 6.07156E-19 |
| rs12666117 | A | G | 0.0274011 | 0.0048 | 1.34602E-08 |
| rs12958048 | G | A | -0.0338022 | 0.0051 | 3.61327E-11 |
| rs1354115 | A | C | 0.0275957 | 0.0049 | 2.37001E-08 |
| rs1432639 | A | C | 0.0389995 | 0.005 | 4.55302E-15 |
| rs159963 | A | C | -0.0270013 | 0.0049 | 3.19301E-08 |
| rs17727765 | C | T | 0.0507987 | 0.0088 | 8.51295E-09 |
| rs1806153 | T | G | 0.0361005 | 0.0059 | 1.17801E-09 |
| rs2005864 | T | C | 0.0281987 | 0.0049 | 6.73101E-09 |
| rs2389016 | T | C | 0.0305001 | 0.0053 | 1.01801E-08 |
| rs247910 | G | A | 0.031501 | 0.0049 | 1.065E-10 |
| rs34215985 | G | C | 0.037297 | 0.0063 | 3.13199E-09 |
| rs4074723 | C | A | 0.0270013 | 0.0049 | 3.11803E-08 |
| rs4904738 | C | T | 0.0289037 | 0.0049 | 2.57199E-09 |
| rs5758265 | A | G | 0.0310044 | 0.0054 | 7.55405E-09 |
| rs61867293 | T | C | -0.0374008 | 0.0061 | 6.96594E-10 |
| rs6905391 | A | G | -0.0442968 | 0.0069 | 1.348E-10 |
| rs7198928 | C | T | -0.0284028 | 0.005 | 1.00399E-08 |
| rs7430565 | A | G | -0.0288008 | 0.0048 | 2.86801E-09 |
| rs7856424 | T | C | -0.0306035 | 0.0053 | 8.47891E-09 |
| rs8025231 | C | A | 0.0338981 | 0.0048 | 2.35776E-12 |
| rs8063603 | A | G | -0.0307995 | 0.0053 | 6.86499E-09 |
| rs915057 | G | A | 0.0299954 | 0.0049 | 7.60904E-10 |
| rs9402472 | A | G | 0.0326995 | 0.0059 | 2.78099E-08 |
| rs9427672 | G | A | 0.0320997 | 0.0058 | 3.11903E-08 |
| ***SNPs that predicted the cortical surface area of banks of the superior temporal sulcus*** | | | | | |
| rs7862092 | T | G | 8.8051 | 1.5998 | 3.713E-08 |
| ***SNPs that predicted the function of causal anterior cingulate*** | | | | | |
| rs10845985 | A | G | 3.971 | 0.7635 | 1.982E-07 |
| ***SNPs that predicted the function of entorhinal cortex*** | | | | | |
| rs3171927 | A | G | -2.4501 | 0.522 | 2.688E-06 |
| ***SNPs that predicted the function of inferior parietal lobule*** | | | | | |
| rs7862092 | T | G | 30.4563 | 6.106 | 6.104E-07 |
| ***SNPs that predicted the function of lateral occipital cortex*** | | | | | |
| rs2277499 | T | G | -14.7967 | 3.062 | 1.35E-06 |
| ***SNPs that predicted the function of lateral orbitofrontal cortex*** | | | | | |
| rs4897178 | T | G | -12.5712 | 1.4564 | 6.034E-18 |
| ***SNPs that predicted the function of lingual cortex*** | | | | | |
| rs6496265 | C | G | 11.6294 | 2.5419 | 4.759E-06 |
| ***SNPs that predicted the function of medial orbitofrontal cortex*** | | | | | |
| rs9375435 | T | C | 5.4697 | 1.0458 | 1.694E-07 |
| ***SNPs that predicted the function of middle temporal cortex*** | | | | | |
| rs62256903 | A | G | -9.5852 | 1.874 | 3.14E-07 |
| ***SNPs that predicted the function of parahippocampal cortex*** | | | | | |
| rs1792354 | T | C | -3.7416 | 0.6234 | 1.955E-09 |
| rs58131984 | T | G | 3.5557 | 0.662 | 7.841E-08 |
| ***SNPs that predicted the function of pericalcarine cortex*** | | | | | |
| rs6461386 | A | G | 11.0645 | 1.6148 | 7.279E-12 |
| ***SNPs that predicted the function of postcentral cortex*** | | | | | |
| rs11789773 | A | C | -17.2183 | 2.911 | 3.32E-09 |
| rs34322452 | A | G | 19.845 | 2.5959 | 2.093EE-14 |
| ***SNPs that predicted the function of posterior cingulate cortex*** | | | | | |
| rs11695609 | T | C | -5.5681 | 0.8688 | 1.465E-10 |
| ***SNPs that predicted the function of rostral middle frontal cortex*** | | | | | |
| rs4670555 | T | C | 15.7039 | 3.0529 | 2.691E-07 |
| ***SNPs that predicted the global cortical thickness*** | | | | | |
| rs2636563 | C | G | 0.0044 | 9E-04 | 2.299E-06 |
| ***SNPs that predicted the cortical thickness of caudal middle frontal cortex*** | | | | | |
| rs199441 | A | G | -0.0043 | 9E-04 | 4.973E-06 |
| ***SNPs that predicted the cortical thickness of pars triangularis*** | | | | | |
| rs1979035 | A | G | 0.004 | 9E-04 | 2.131E-06 |
| ***SNPs that predicted the cortical thickness of posterior cingulate*** | | | | | |
| rs10117809 | T | C | 0.0074 | 0.0014 | 2.163E-07 |
| ***SNPs that predicted the cortical thickness of superior temporal cortex*** | | | | | |
| rs199441 | A | G | -0.0059 | 0.001 | 1.646E-08 |
| rs62515046 | T | G | -0.0044 | 9E-04 | 4.566E-07 |
| ***SNPs that predicted the cortical thickness of transverse temporal gyrus*** | | | | | |
| rs68044532 | T | C | 0.0097 | 0.0019 | 3.344E-07 |

**Abbreviations:** MDD, major depressive disorder. SNP, single nucleotide polymorphism.

**Footnote:** In the Mendelian randomization analysis of MDD on cortical surface area and thickness, the SNPs were selected at a p-value cut-off point of 5e-8. In the analysis of cortical surface area and thickness on MDD, the SNPs were selected at a p-value cut-off point of 5e-6 because no SNPs were selected by the standard. We tried relaxing the standard to 5e-5, but the number of SNPs did not increase. When the standard was relaxed to 5e-4, the number of SNPs did not exceed 5. Because of the risk of weak instrument bias, we continued to use the standard of 5e-6.

# Supplementary Figure 1. Forest plots for leave one out analysis


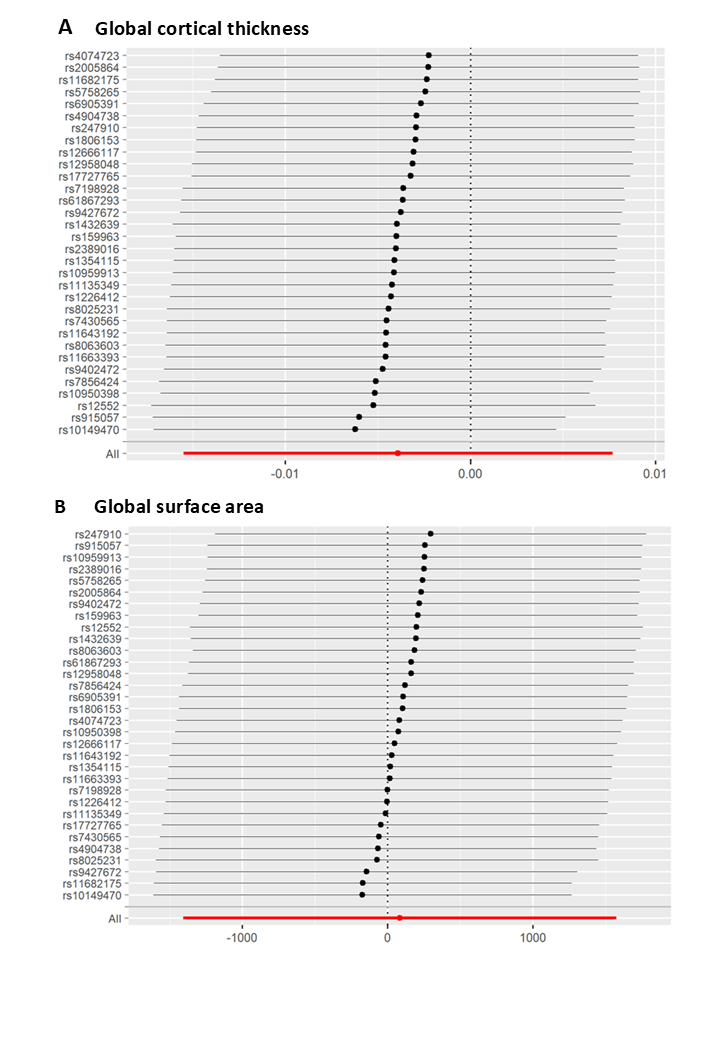


**Footnote:** The figure shows the contribution to the effect of MDD on global cortical thickness (figure 1A, the top half) and global surface area (figure 1B, the bottom half).
